# Molecular Insights into the Interaction of Orexin 1 Receptor Antagonists: A Comprehensive Study Using Classical and Quantum Computational Methods

**DOI:** 10.3390/molecules30132790

**Published:** 2025-06-28

**Authors:** Caio Sena, Pedro Albuquerque, Jonas Oliveira, Davi Vieira

**Affiliations:** 1 Instituto de Química, Universidade Federal do Rio Grande do Norte, Natal 59072-970, RN, Brazil; pedro.albuquerque.087@ufrn.edu.br; 2 Departamento de Biofísica e Farmacologia, Universidade Federal do Rio Grande do Norte, Natal 59072-970, RN, Brazil; jonasivan@gmail.com

**Keywords:** orexin 1 receptor, orexin receptor antagonists, molecular dynamics simulation, molecular fractionation with conjugate caps, density functional theory, sleep disorders, drug–receptor interactions

## Abstract

Sleep disorders, such as insomnia and narcolepsy, significantly impact quality of life. They are often associated with long-term health consequences, including cardiovascular disease, immune dysfunction, and cognitive impairment. While traditional treatments, such as sedatives and hypnotics, can be effective, they are limited by issues of tolerance and dependence. The orexinergic system, particularly the orexin 1 receptor (OXR1), has emerged as a promising therapeutic target due to its central role in regulating sleep–wake cycles. In this study, we investigate the molecular interactions of three OXR1 antagonists—daridorexant, lemborexant, and suvorexant—using an integrated computational approach combining molecular dynamics (MD) simulations, density functional theory (DFT) calculations, and the molecular fractionation with conjugate caps (MFCC) methodology. The MFCC approach enabled the precise quantification of interaction energies between ligands and key receptor residues, providing detailed insights into the contributions of specific amino acids to binding stability. Our results reveal that residues such as GLU204, HIS216, and ASN318 play critical roles in stabilizing ligand–receptor interactions, with a marked decrease in binding energy magnitude as dielectric constants increase. Daridorexant exhibited the strongest interaction energy, driven by hydrogen bonds and hydrophobic contacts, while lemborexant and suvorexant showed distinct stabilization patterns mediated by hydrophobic interactions. These findings provide a robust molecular basis for the rational design of next-generation OXR1 antagonists with improved efficacy and safety profiles. By elucidating drug–receptor interactions at the atomic level, this research underscores the impact of integrated computational approaches in drug discovery. It supports the development of precise targeted therapies for sleep disorders.

## 1. Introduction

A detailed understanding of the molecular interactions between drugs and receptors is fundamental to the development of new, more effective, and safer medications. These interactions, which occur at the atomic level, determine the affinity, selectivity, and stability of drug–receptor complexes. These parameters directly influence therapeutic efficacy and reduce side effects. In this context, the use of advanced computational methods, such as molecular docking, molecular dynamics simulations, and quantum calculations, has proven to be essential for unraveling the molecular mechanisms governing these interactions, enabling rational optimization of compounds with pharmacological potential [1,2,3,4,5].

The orexinergic system, composed of the neuropeptides orexin A and B and their G-protein-coupled receptors (OXR1 and OXR2) [6,7], plays a crucial role in regulating various physiological functions, which, in turn, contribute to overall homeostatic balance [8]. Dysfunction of this system is associated with a variety of disorders. Sleep-related disorders such as insomnia and narcolepsy have been strongly linked to orexin system impairment [9,10]. Metabolic disorders like obesity [11] and neurodegenerative diseases such as Parkinson’s and Alzheimer’s [12,13,14] have also been implicated. Sleep disorders in particular encompass a wide range of conditions that impair both the quality and quantity of sleep, significantly affecting daily functioning and overall health. Common sleep disorders include insomnia, narcolepsy, sleep apnea, restless legs syndrome, and circadian rhythm disturbances [15]. Sleep disorders are often linked to orexinergic system dysregulation and exacerbation by conditions such as stress, anxiety [16], or other physical ailments. Prolonged disturbances can lead to serious long-term consequences, including increased risk of cardiovascular disease [17,18,19], impaired immunity [20,21], and cognitive decline [22,23].

Traditionally, insomnia has been treated with medications such as sedatives, hypnotics, and Z-drugs, which aim to modulate the sleep-promoting (e.g., GABAergic) and wakefulness-promoting (e.g., histaminergic) systems. Despite their effectiveness, these drugs are often associated with substantial limitations, notably tolerance and severe dependence, which can profoundly disturb the sleep–wake cycle [24,25,26]. In light of these limitations, there is a critical need to pursue safer and more targeted therapeutic strategies. In this context, while dual orexin receptor antagonists (DORAs) modulate both receptors to induce physiological sleep, recent evidence highlights OXR1 as an emerging target for behavioral disorders. Studies show a crucial role in anxiety, where it regulates stress responses and avoidance behaviors [27], and in chemical dependence, where it controls dopamine release [28]. In addition, its modulation has shown antidepressant effects in animal models [29]. This dual function—the regulation of sleep in the lateral hypothalamus and emotional modulation in limbic circuits—positions OXR1 as a key target for multifocal therapeutic approaches.

In this study, we investigate the molecular interactions of three dual orexin receptor antagonists with OXR1—daridorexant [30,31], lemborexant [32,33], and suvorexant [34,35]. We use an integrated approach that combines classical molecular docking and molecular dynamics (MD) simulations to explore dynamic ligand–receptor contacts. Additionally, the molecular fractionation with conjugate caps (MFCC) methodology and quantum calculations are employed to quantify interaction energies between ligands and receptor amino acid residues. Through this comprehensive analysis, we aim to elucidate the structural and energetic details governing the complexation of these compounds with OXR1, including critical residues and binding affinities.

## 2. Results

### 2.1. Ligand Optimization

The structures of the three ligands were obtained from the PubChem database and subjected to thorough optimization. Initially, the geometries underwent optimization using a Dreiding-like force field, followed by energy minimization with the CHARMm force field, version c44b2, in BIOVIA Discovery Studio, version 2022. The SmartMinimizer algorithm was applied in a solvent environment featuring a distance-dependent dielectric.

Conformational space analysis revealed that daridorexant exhibited fifteen unique conformations, indicating moderate structural flexibility, while lemborexant presented nine distinct conformations, suggesting slightly lower flexibility. In contrast, suvorexant exhibited only a single conformation, highlighting its structural rigidity. The radius of gyration was measured at 4.20 Å for daridorexant and 4.44 Å for lemborexant, indicating a slightly more elongated structure for the latter. Because of the single conformation of suvorexant, an average radius of gyration was not determined. The average solvent-accessible surface area (SASA) values for daridorexant and lemborexant were 643.00 and 653.59 Å^2^, respectively, reflecting their potential to interact with the surrounding solvent. Daridorexant exhibited root-mean-square deviation (RMSD) values ranging from 0.21 Å to 4.52 Å (mean: 2.71 Å). For lemborexant, the RMSD values were 0.90, 2.97, and 2.05 Å. Suvorexant, having only one conformation, did not exhibit measurable variations in RMSD.

### 2.2. Quantum Energies and Quantum Chemical Descriptors of Ligands

The most stable conformer of each ligand, obtained in the previous stage, was subjected to a second stage of optimization using quantum mechanical calculations. Quantum chemical calculations focusing on molecular orbitals (MOs) and density functional theory (DFT), employing the generalized gradient approximation (GGA) exchange–correlation functional PBE and the 6-311++G basis set [36], were performed to optimize the ligand geometries and analyze their electronic properties. The optimized structures were then used in docking and molecular dynamics simulations to evaluate the binding affinities and investigate the dynamic behavior of the ligand–receptor complexes.

**Figure 1:**
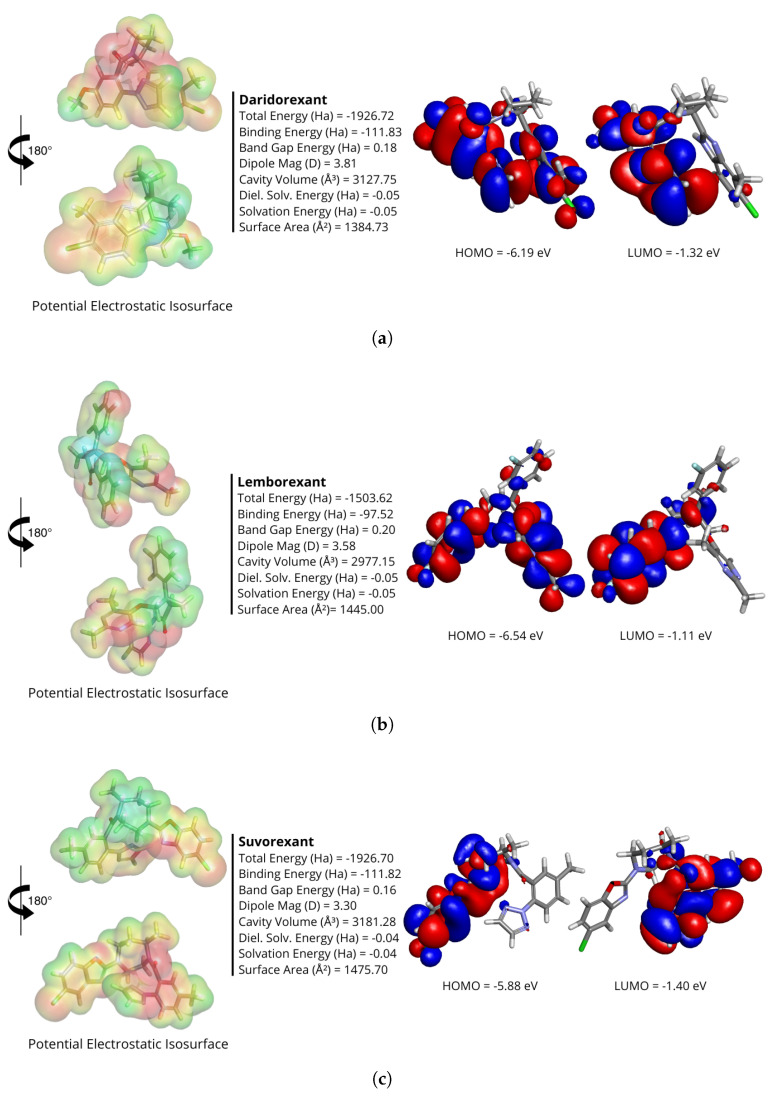
Quantum energies and electrostatic properties of daridorexant (**a**), lemborexant (**b**), and suvorexant (**c**). Properties displayed are total energy, binding energy, HOMO energy, LUMO energy, band gap energy, dipole magnitude, cavity volume, dielectric energy, solvation energy, and surface area. The figure presents 3D visualizations of the potential electrostatic isosurface (**left panel**) and the HOMO and LUMO structures (**right panel**).

The potential electrostatic isosurface of all the optimized structures, illustrated in Figure 1, provides insights into the distribution of positive and negative charges and regions with varying electron densities. The negative (red) regions are concentrated around electronegative atoms (O and N), while the positive (blue) regions are primarily centered on carbon atoms.

The total energies were −1926.72 Ha for daridorexant, −1503.62 Ha for lemborexant, and −1926.70 Ha for suvorexant. These absolute values reflect the internal electronic energy of each molecule under the selected computational conditions (GGA-PBE/6-311++G) and are not directly comparable across molecules with different sizes and compositions. Nevertheless, the negative values are consistent with stable ground-state configurations. The binding energies were −111.83 Ha for daridorexant, −97.52 Ha for lemborexant, and −111.82 Ha for suvorexant. These negative values indicate that the formation of the ligand structures is energetically favorable. The dipole moments were 3.81 D for daridorexant, 3.58 D for lemborexant, and 3.30 D for suvorexant. These values indicate differences in charge distribution, which may affect electrostatic interactions with the receptor. The cavity volumes were 3127.75 Å^3^ for daridorexant, 2977.15 Å^3^ for lemborexant, and 3181.28 Å^3^ for suvorexant. The corresponding dielectric solvation energies, calculated to assess the energy change from vacuum to solvent, were −0.0484 Ha, −0.0518 Ha, and −0.0400 Ha, respectively. The calculated surface areas were 1384.73 Å^2^ for daridorexant, 1445.00 Å^2^ for lemborexant, and 1475.70 Å^2^ for suvorexant, highlighting the extent of their potential interactions with the environment. The differences in cavity volume and surface area indicate the varied accessibility and binding potential of these ligands to the receptor site.

The highest occupied molecular orbital (HOMO) and lowest unoccupied molecular orbital (LUMO) energy levels, along with quantum chemical descriptors such as chemical hardness (η), softness (σ), chemical potential (μ), electronegativity index (χ), and electrophilicity index (ω), were determined for the ligands and are presented in Table 1. The HOMO energy values were −6.19316 eV, −6.53966 eV, and −5.87751 eV, respectively, while the corresponding LUMO energy values were −1.32595 eV, −1.11510 eV, and −1.40361 eV. For daridorexant, lemborexant, and suvorexant, the calculated HOMO–LUMO energy gaps were 4.86721 eV, 5.42455 eV, and 4.47390 eV, respectively. Figure 1 illustrates the HOMO and LUMO for the ligands. It is important to note that the values of quantum descriptors obtained by DFT, such as the HOMO–LUMO gap, depend on the choice in the exchange–correlation functional. Moreover, DFT calculations tend to underestimate the band gap compared to experimental data, especially when using GGA functionals such as PBE.

### 2.3. Molecular Docking

**Figure 2:**
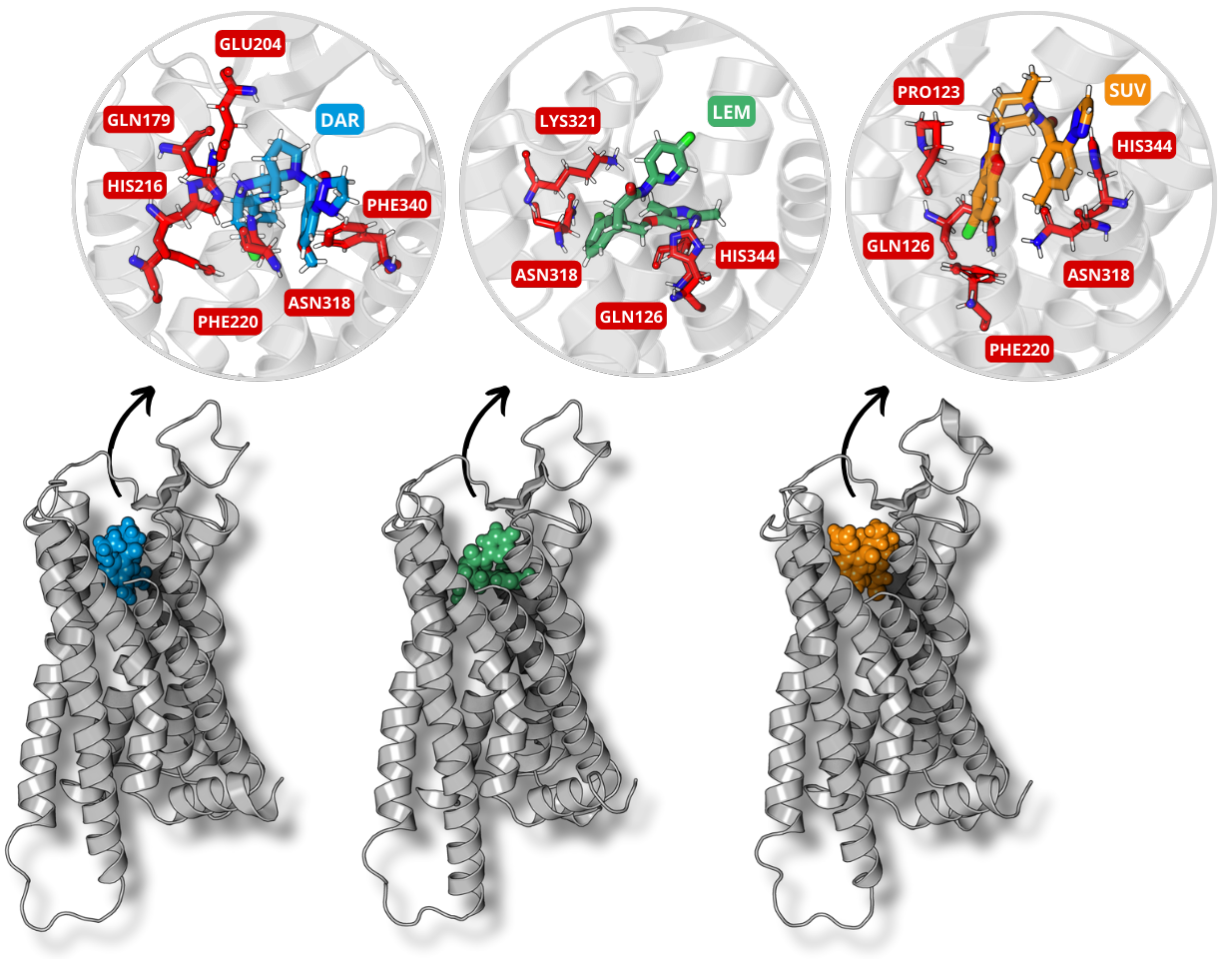
Representation of the most stable conformational structures of DORA–OXR1 complexes obtained as a result of molecular docking for daridorexant (blue), lemborexant (green), and suvorexant (orange). The receptor is shown in ribbon with ligands regarding the surface style. Ligands in the interaction pocket are emphasized in stick style.

After the reconstruction of the model, the final structure underwent molecular docking studies aimed at assessing the interaction between the reconstructed receptor and the ligands daridorexant, lemborexant, and suvorexant. This strategy enabled the acquisition of stable complexes between the compounds and the receptor, determining the most favorable binding sites on the basis of binding energy scores and preferred conformations, determined by the scoring function of HADDOCK 2.4 (Table 2). Along with the HADDOCK score (HS), the results present the number of structures per cluster, RMSD, relative energy of van der Waals interactions (E_VDW_), Coulomb electrostatics (E_ELEC_), and the Z-score for each DORA–OXR1 complex. The energetically most favorable conformations, derived from molecular docking studies, were selected for subsequent molecular dynamics simulations to elucidate their stability and interaction characteristics. The structures of these optimized complexes are shown in Figure 2, which illustrates the receptor in a ribbon representation and the ligands within their binding pockets in a surface representation, with the ligand highlighted in a stick format to emphasize its positioning within the interaction site.

### 2.4. Dynamics and Intermolecular Interactions of DORA–OXR1 Complexes

**Figure 3:**
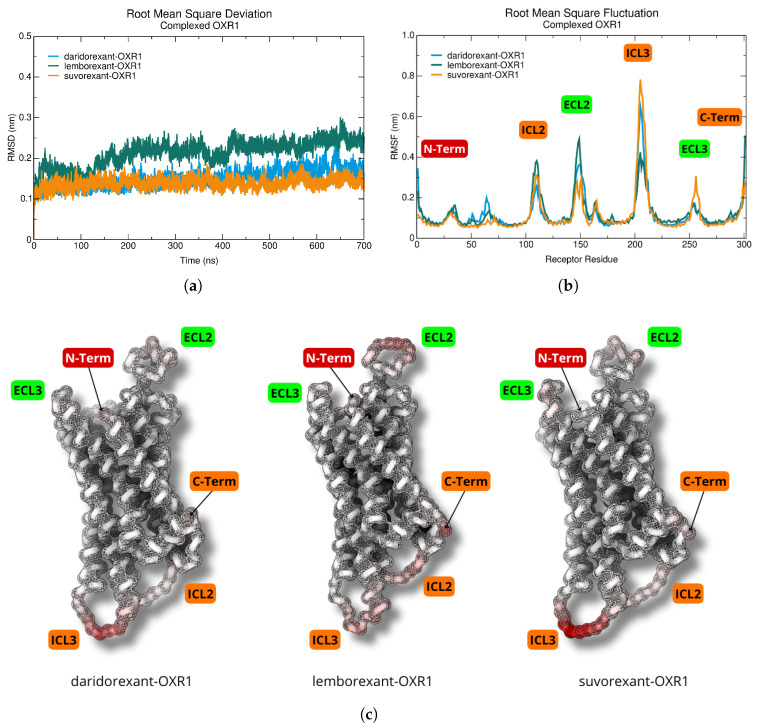
Dynamics and structural flexibility for DORA–OXR1 complexes analyzed over a 700 ns molecular dynamics simulation. (**a**) The root-mean-square deviation (RMSD) plot. (**b**) Root-mean-square fluctuation (RMSF) plot highlights flexibility across residues, with higher fluctuations in terminal regions (N-term and C-term), extracellular (ECL) and intracellular (ICL) loops, and solvent-exposed areas. (**c**) B-factor structure representation in surface and cartoon highlighting the atomic displacement within the OXR1 receptor. B-factor coloring to indicate regions of varying flexibility. The red areas represent residues with higher B-factors (terminals and loops), signifying greater flexibility, while white areas represent more rigid regions (particularly around the binding site).

We employed molecular dynamics simulations to determine receptor–drug binding affinities, reveal unique structural properties, and categorize the binding sites according to their conformational stability. This study examined the behavior of the orexin 1 receptor in complex with daridorexant, lemborexant, and suvorexant using 700 ns simulations. The RMSD was calculated for the α-carbons of the OXR1 receptor over the total simulation time, Figure 3a, revealing distinct stabilization patterns among the complexes. The daridorexant–OXR1 and suvorexant–OXR1 complexes exhibited early stabilization, indicating a faster convergence to an equilibrium structure, while the lemborexant–OXR1 complex initially showed pronounced fluctuations, stabilizing only after approximately 150 ns, indicating slower conformational convergence. The root-mean-square fluctuation (RMSF), calculated for the α-carbons of OXR1, Figure 3b, showed that most residues maintained low fluctuation values (≈0.2 nm), consistent with a relatively rigid structure. However, higher fluctuations were observed in extracellular and intracellular loops, terminal ends, and solvent-exposed regions, suggesting increased molecular flexibility. The residues surrounding the binding pocket exhibited lower RMSF values, highlighting the stabilizing influence of ligand interactions.

The B-factor, a measure of atomic displacement, as shown in Figure 3c, corroborated these findings, illustrating the parameters of atomic displacement. The red regions indicate increased flexibility, while lighter hues denote structurally rigid regions. Ligands remained stably positioned within the binding site, reinforcing the rigidity observed in these critical regions. However, intracellular loops ICL2 and ICL3, as well as extracellular loops ECL2 and ECL3, exhibited increased flexibility, similar to the behavior observed in the C-terminal domain. In particular, the ICL3 loop exhibited significant mobility in the suvorexant–OXR1 complex, while, in the lemborexant–OXR1 complex, the most pronounced dynamic movements occurred in the C-terminal region and the ECL2 loop, suggesting enhanced molecular flexibility. Overall, the molecular dynamics simulations revealed distinct dynamic profiles for each ligand–receptor complex, highlighting variations in their conformational stability and flexibility.

The final configurations from the molecular dynamics simulations of the DORA–OXR1 complexes were used for an exhaustive quantitative assessment of the energetic roles played by specific amino acid residues involved in interactions with ligand compounds. This analysis employed the MFCC method for precise interaction energy quantification between ligands and amino acid residues. Energetic contributions were ascertained via single-point energy calculations on fixed molecular geometries using DFT with the B97D functional and the 6-311+G(d,p) basis set.

The interaction energies of the three ligands were separately evaluated within an 8 Å radius of individual binding pocket regions. Convergence criteria were established by examining the distance to receptor residues. Amino acid residues affected by attractive and repulsive forces were considered to precisely describe these interactions. The total interaction energy was calculated by summing up the contributions from individual residues, ensuring energy convergence. The optimal binding pocket radius was determined when further expansion resulted in interaction energy changes below 10%.

**Figure 4:**
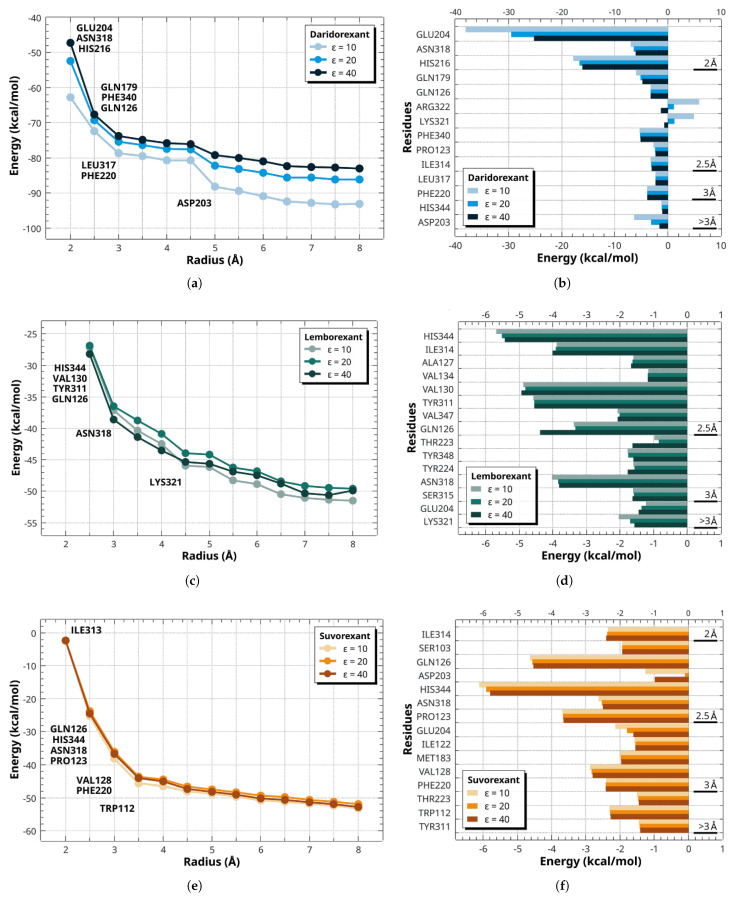
Graphical representation of the total interaction energy (kcal/mol) as a function of the binding pocket radius (Å) and the most relevant amino acid contributions in the interaction energy of the DORA–OXR1 complexes in the pocket binding. Panels (**a**,**b**) correspond to daridorexant (blue), (**c**,**d**) to lemborexant (green), and (**e**,**f**) to suvorexant (orange). Interaction energies were calculated using the GGA B97D functional within the MFCC framework under different dielectric constants ϵ (10, 20, and 40).

Figure 4 presents the energy profiles for the OXR1 receptor in complex with DORAs. At dielectric constants ϵ of 10, 20, and 40, daridorexant showed significantly stronger interaction energies (kcal/mol) with OXR1 (−93.05, −86.18, and −83.02) compared to lemborexant (−51.47, −49.58, and −49.87) and suvorexant (−53.18, −51.94, and −52.77). For daridorexant, Figure 4a,b, significant variations in interaction energy were observed between 2.0 and 3.0 Å, primarily due to interactions with residues GLU204, ASN318, and HIS216 at 2.0 Å; GLN179, PHE340, and GLN126 at 2.5 Å; and LEU317 and PHE220 at 3.0 Å. A more abrupt energy fluctuation was noted at 5.0 Å, attributed to ASP203, beyond which the energy stabilized. In the case of lemborexant, Figure 4c,d, energy fluctuations were most pronounced up to 3.0 Å, influenced by HIS344, VAL130, TYR311, and GLN126, with progressive stabilization occurring between 4.0 and 6.0 Å. Notably, ASN318 and LYS321 contributed significantly at 3.0 and 4.5 Å, respectively. For suvorexant, Figure 4e,f show that the largest fluctuations were observed between 2.0 and 3.0 Å, corresponding to interactions with ILE313 at 2.0 Å; GLN126, HIS344, ASN318, and PRO123 at 2.5 Å; VAL128 and PHE220 at 3.0 Å; and TRP112 at 3.5 Å, after which the energy stabilized. The spatial arrangement of binding site residues governs ligand interactions; thus, defining their role is essential for elucidating receptor–ligand complex functional diversity.

For the daridorexant–OXR1 complex, Figure 4b, the most significant residues, in descending order of interaction energy (kcal/mol), include GLU204 (−25.18), HIS216 (−16.04), ASN318 (−6.04), PHE340 (−5.13), GLN179 (−4.74), PHE220 (−3.83), GLN126 (−3.23), ILE314 (−3.01), PRO123 (−2.33), LEU317 (−2.26), ASP203 (−1.57), ARG322 (−1.26), HIS344 (−1.00), and LYS321 (−0.68).

In the lemborexant–OXR1 complex, Figure 4d, the most relevant residues, in descending order of interaction energy (kcal/mol), include HIS344 (−5.43), VAL130 (−4.93), TYR311 (−4.54), GLN126 (−4.38), ILE314 (−4.00), ASN318 (−3.81), VAL347 (−2.07), TYR244 (−1.77), ALA127 (−1.66), SER315 (−1.63), THR223 (−1.63), TYR348 (−1.59), LYS321 (−1.56), GLU204 (−1.44), and VAL134 (−1.17).

For the suvorexant–OXR1 complex, Figure 4f, the most significant residues, in descending order of interaction energy (kcal/mol), include HIS344 (−5.79), GLN126 (−4.52), PRO123 (−3.65), VAL128 (−2.78), ASN318 (−2.49), PHE220 (−2.41), ILE314 (−2.39), TRP112 (−2.27), MET183 (−1.96), SER103 (−1.92), GLU204 (−1.60), ILE122 (−1.54), THR223 (−1.44), TYR311 (−1.40), and ASP203 (−0.98).

**Figure 5:**
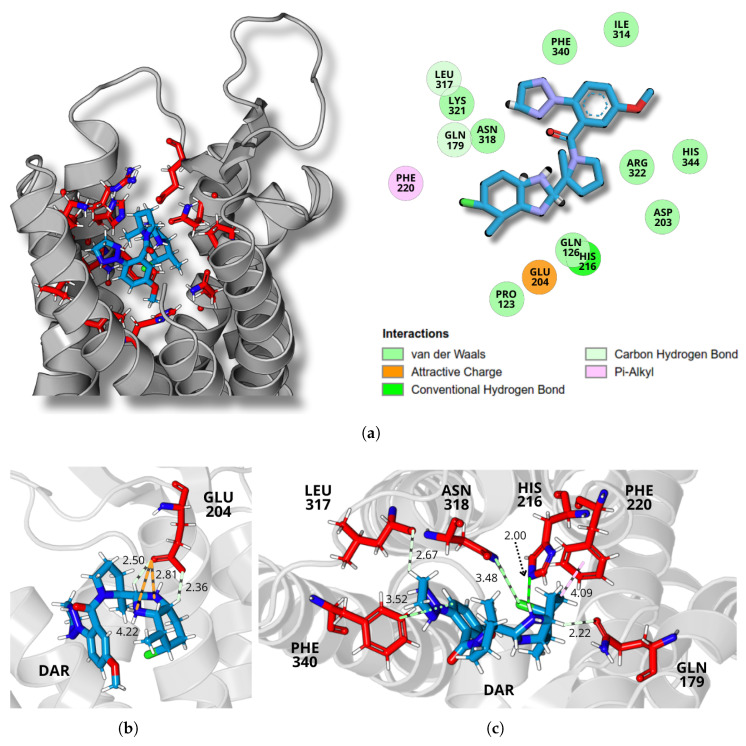
A detailed depiction of daridorexant’s interactions at the OXR1 binding site. (**a**) 3D representation of the receptor in ribbon format, with the binding site residues (red) and ligand (blue) shown in stick representation. The adjacent 2D diagram highlights key ligand–receptor interactions, categorized by interaction type. (**b**) Intermolecular interactions between daridorexant and GLU204 amino acid. (**c**) Intermolecular interactions between daridorexant and GLN179, HIS216, PHE220, LEU317, ASN318, and PHE340 amino acids. Dashed lines refer to attractive charge interactions (orange), carbon–hydrogen bonds (light green), conventional hydrogen bonds (dark green), halogen interactions, alkyl interactions (green), and π–alkyl interactions (pink).

**Figure 6:**
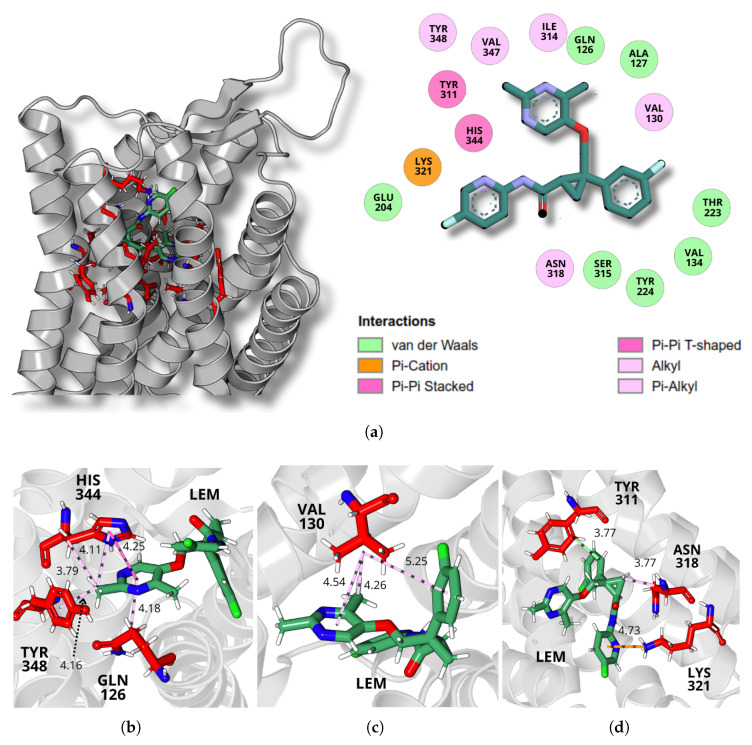
A detailed depiction of lemborexant’s interactions at the OXR1 binding site. (**a**) 3D representation of the receptor in ribbon format, with the binding site residues (red) and ligand (green) shown in stick representation. The adjacent 2D diagram highlights key ligand–receptor interactions, categorized by interaction type. (**b**) Intermolecular interactions between lemborexant and GLN126, TYR348, and HIS344 amino acids. Dashed lines refer to π–alkyl interactions (light pink) and π–π T-shaped interaction (pink). (**c**) Intermolecular interactions between lemborexant and VAL130 amino acid. Dashed lines refer to alkyl and π–alkyl interactions (light pink). (**d**) Intermolecular interactions between lemborexant and TYR311, ASN318, and LYS312 amino acids. Dashed lines refer to van der Waals interaction (light green), π–cation interaction (orange), and alkyl interaction (pink).

**Figure 7:**
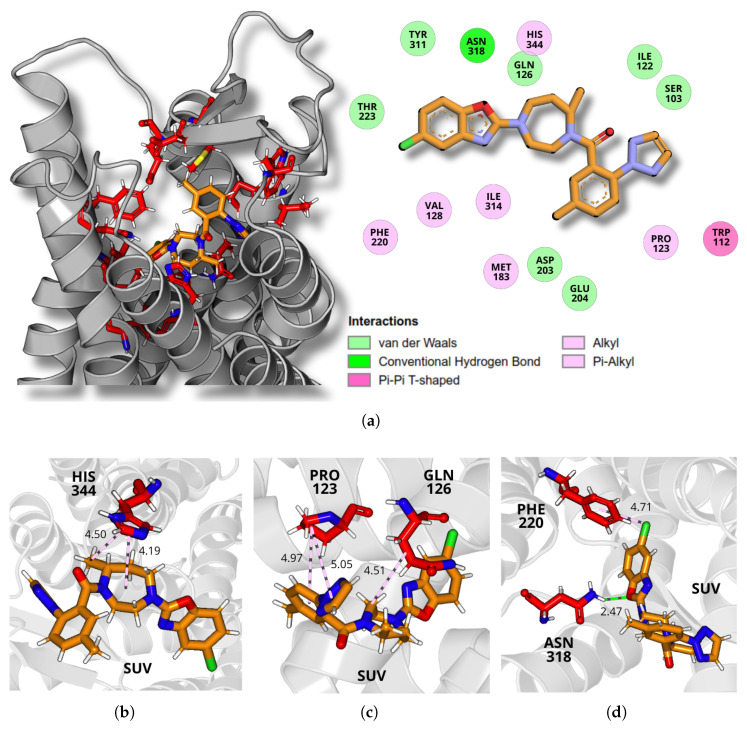
A detailed depiction of suvorexant’s interactions at the OXR1 binding site. (**a**) 3D representation of the receptor in ribbon format, with the binding site residues (red) and ligand (orange) shown in stick representation. The adjacent 2D diagram highlights key ligand–receptor interactions, categorized by interaction type. (**b**) Intermolecular interactions between suvorexant and HIS344 amino acid. Dashed lines refer to π–alkyl interactions (light pink). (**c**) Intermolecular interactions between suvorexant and PRO123 and GLN126 amino acids. Dashed lines refer to alkyl and π–alkyl interactions (light pink). (**d**) Intermolecular interactions between suvorexant and PHE220 and ASN318 amino acids. Dashed lines refer to π–alkyl interaction (light pink) and conventional hydrogen bond (dark green).

A detailed analysis of the final molecular dynamics simulation structure revealed the specific intermolecular interactions between the ligands and the receptor binding site, illustrated in Figure 5, Figure 6 and Figure 7, for daridorexant, lemborexant, and suvorexant, respectively. The panel (a) of each figure presents the receptor structure in ribbon format, highlighting the residues that constitute the binding pocket, with the ligand represented in sticks. A 2D interaction map illustrates residue contacts within 5 Å of the ligand in the binding pocket, highlighting buried interactions and their classifications. The analysis of the interactions reveals the presence of both classical and non-classical hydrogen bonds, as well as hydrophobic interactions, including alkyl and π–alkyl types.

For daridorexant, stabilization occurs through interactions with residues GLN179, GLU204, HIS216, PHE220, LEU317, ASN318, and PHE340. The residue GLU204 establishes interactions with daridorexant through its carbonyl group, forming two distinct types of bonds, as shown in Figure 5b. Two key charge interactions are identified in daridorexant’s binding: GLU204’s OE1 atom interacts with the ligand’s N2 and N3 atoms at 2.81 Å and 4.22 Å, respectively. Complementing these, two carbon–hydrogen bonds (nonconventional H-bonds) further stabilize the complex, involving GLU204’s OE2 with daridorexant’s H17 (2.36 Å) and GLU204’s OE1 with H16 (2.50 Å). Notably, the charge interactions appear to be crucial for daridorexant’s stable binding.

Through the amide group of its side chain, specifically the oxygen atom, the glutamine amino acid GLN179 forms a carbon–hydrogen bond, where its OE1 atom interacts with the H17 atom of the ligand at a distance of 2.22 Å. Similarly, LEU317 establishes a carbon–hydrogen bond, with its backbone carbonyl oxygen atom interacting with the H31 atom of daridorexant at 2.67 Å. Additionally, HIS216 contributes to the stabilization of the ligand by forming a conventional hydrogen bond, in which its NE2 atom, the saturated nitrogen of the aromatic side chain of the imidazole group, interacts with the H33, the hydrogen atom bound to the nitrogen NS2 of an amino group of the ligand at a distance of 2.00 Å, as illustrated in Figure 5c.

Furthermore, additional interactions contribute to the stabilization of daridorexant within the binding pocket. The amino acid asparagine, residue ASN318, forms a halogen (chlorine) interaction, where its ND2 atom of the amide group interacts with the Cl atom of the ligand at a distance of 3.48 Å. Moreover, PHE220 engages in a π–alkyl interaction with the ligand, with a contact distance of 2.22 Å. Similarly, PHE340 participates in an alkyl interaction with the ligand, positioned at a distance of 3.52 Å, as illustrated in Figure 5c. Together, these interactions reinforce the accommodation of the ligand within the receptor site, enhancing the overall stability of the complex.

For lemborexant, stabilization is mainly driven by interactions with the residues GLN126, VAL130, TYR311, ASN318, LYS321, HIS344, and TYR348. The HIS344 residue establishes three key interactions, as depicted in Figure 6b. The first is a π–π stacking interaction at a distance of 4.25 Å, where the π-electrons of the imidazole group in HIS344 interact with the π-electrons of the pyrimidine ring of the ligand, composed of atoms N1, C14, N2, C15, C12, and C13. The second is a π–alkyl interaction at 4.11 Å, involving the π-electrons of the HIS344 ring and the C21 atom of lemborexant. Additionally, HIS344 forms an alkyl interaction at 4.11 Å between its CB carbon atom and the C21 carbon atom of the ligand.

Furthermore, as shown in Figure 6b, TYR348 engages in a π–alkyl interaction with lemborexant, where the phenol ring of its side chain interacts with the C21 carbon atom at 4.16 Å. The residue GLN126 also participates in a π–alkyl interaction at 4.18 Å, where its CG carbon interacts with the π-electrons of the ligand ring (N1, C14, N2, C15, C12, and C13).

As illustrated in Figure 6c, the amino acid valine, residue VAL130, establishes three distinct interactions with the lemborexant molecule through the isopropyl group of its side chain. The first is an alkyl interaction with the C22 atom of the ligand at a distance of 2 Å. Additionally, two π–alkyl interactions contribute to stabilization: one involves the π-electrons of the ligand’s pyrimidine ring (N1, C14, N2, C15, C12, and C13) at 4.54 Å, while the other involves the π-electrons of the ligand’s benzene ring (C6–C11) at 5.25 Å.

Further stabilizing interactions, as depicted in Figure 6d, include a π–cation interaction involving LYS321, where the π-electrons of the ligand’s pyridine ring (N4, C16–C20) interact with the NZ atom of the residue’s side chain at 4.73 Å. Additionally, TYR311 establishes a van der Waals interaction, where the apolar carbon atoms C11 of the ligand and CD1 of the amino acid interact at 3.77 Å. Lastly, ASN318 forms an alkyl interaction in which its CB atom interacts with the C2 carbon atom of the ligand at 3.77 Å. Collectively, these diverse interactions, including π–cation, π–alkyl, van der Waals, and hydrogen bonding, contribute to the robust binding of lemborexant within the receptor.

For suvorexant, stabilization is primarily driven by interactions with residues PRO123, GLN126, PHE220, ASN318, and HIS344. Among these, the HIS344 residue plays a key role by establishing two π–alkyl interactions. In the first, the π-electrons of the HIS344 ring interact with the C23 atom of the ligand at 4.50 Å. In the second, the same π-electrons of the amino acid’s ring interact with the alkyl group of the ligand’s diazepine ring (N1, C8–C9, N2, and C10–C12) at 4.19 Å, as shown in Figure 7b.

As illustrated in Figure 7c, PRO123 establishes two π–alkyl interactions, both mediated by the CD atom of the pyrrolidine group of the residue. These interactions involve the π-electrons of the ligand’s aromatic rings (C14–C19) at 4.97 Å and triazole (C20, C21, N6, N4, and N5) at 5.05 Å. Additionally, GLN126 contributes to stabilization through an alkyl interaction with the C9 carbon atom of the ligand at 4.51 Å.

Further stabilizing interactions include a π–cation interaction between PHE220 and the ligand, where the π-electrons of the residue interact with the Cl atom of the ligand at 4.71 Å. Moreover, ASN318 forms a conventional hydrogen bond, in which its HD22 atom, of the amide group, interacts with the O1 oxygen atom, of the benzoxazole group of the ligand, at 2.47 Å. Collectively, these diverse interactions, especially the π–alkyl interactions involving HIS344 and PRO123, play a crucial role in the stable binding of suvorexant within the receptor binding site.

## 3. Discussion

We used an integrated computational approach to analyze how DORAs bind to OXR1. This provided insights into their mechanisms and therapeutic relevance.

### 3.1. Conformational Analysis of Ligands and Electrostatic Potential Isosurface

The observed conformational flexibility of daridorexant and lemborexant suggests their ability to adopt multiple stable forms, potentially enhancing their adaptability within the receptor binding site. The restricted conformational space of suvorexant, on the other hand, may indicate a highly specific binding mode. The SASA values suggest that daridorexant and lemborexant have comparable solvent-exposed surface areas, which could translate into similar solubility and receptor interaction capabilities. The rigidity of suvorexant might limit its adaptability but enhance its binding specificity.

The RMSD analysis further supports these observations as daridorexant exhibited the highest conformational variability, aligning with its greater pharmacophoric diversity. Lemborexant demonstrated moderate flexibility, suggesting a more stable binding profile, while suvorexant’s lack of RMSD variation confirms its rigid conformation and likely highly specific interaction mode. These findings provide valuable insights into how structural dynamics influence ligand–receptor interactions, with potential implications for optimizing orexin receptor antagonists.

The substantially negative binding energies of daridorexant and suvorexant imply highly stable structures, enhancing interactions with biological targets. Lemborexant’s band gap is the highest, indicating slightly reduced electronic reactivity compared with the other ligands, and it may affect interaction strength and binding affinity. The analysis of dipole moments shows that daridorexant, which has the greatest dipole magnitude, displays considerable charge separation, which affects its solubility and interactions with solvents and biological macromolecules. The larger cavity volumes for daridorexant and suvorexant suggest a higher ability to facilitate binding interactions, possibly improving binding affinity and stability. The solvation energies point to the relative environmental stability of the ligands, with lemborexant having the lowest solvation energy, which could lead to variations in solubility and bioavailability. The molecular surface area of suvorexant is the largest, which could affect its interaction dynamics with receptors and the surrounding environment.

The strongly negative binding energies of daridorexant and suvorexant indicate high structural stability. This may favor interactions with biological targets. The band gap values indicate that lemborexant has the highest value, suggesting slightly lower electronic reactivity compared to the other two ligands. This could influence its interaction strength with the receptor and its potential binding affinity. The dipole moment analysis suggests that daridorexant, with the highest dipole magnitude, exhibits significant charge separation, which can impact its solubility and interactions with solvents and biological macromolecules. The larger cavity volumes observed for daridorexant and suvorexant suggest that these ligands may have an enhanced capacity to accommodate binding interactions with the target receptor, potentially improving their binding affinity and stability. The solvation energies indicate the relative stability of the ligands in different environments, with lemborexant showing the lowest solvation energy, which could translate into differences in solubility and bioavailability. The molecular surface area values suggest that suvorexant has the highest surface exposure, which may influence its interaction dynamics with the receptor and the surrounding environment.

The Electrostatic Potential Isosurface analysis provides further insight into the electrostatic complementarity between the ligands and the receptor binding site. Negative regions near electronegative atoms (O and N) may favor hydrogen bonding, while positive regions on carbon atoms suggest hydrophobic contacts. Together, these features shape the pharmacological profile of the ligands.

### 3.2. Molecular Docking

Molecular docking generated multiple conformations of DORA–OXR1 complexes, with the ligands located at the receptor binding site. Conformations with the lowest HADDOCK scores, indicating high stability, were chosen for the subsequent molecular dynamics simulations to assess their temporal stability and interaction profiles. The docking results demonstrated that all three ligands—daridorexant, lemborexant, and suvorexant—adopted a horseshoe-like conformation within the receptor binding pocket, a structural feature commonly observed in complexed OXR1 antagonists. This conformation improves interactions with key residues within the binding site, such as PRO123 and GLN126 (TM3), HIS216 and PHE220 (TM5), ASN318 (TM6), and HIS344 (TM7), located in transmembrane α-helices essential for receptor activation and stability [37]. Although all the ligands generally adhered to this binding mode, lemborexant showed a subtle difference, placing its pyrimidine ring nearer to the HIS344 (TM7) residue crucial for ligand binding and receptor modulation in G-protein-coupled receptors [38,39].

These findings align with experimental crystallographic studies, which have provided high-resolution structures of OXR1 bound to various antagonists, including lemborexant and suvorexant. The crystal structure of OXR1 in complex with lemborexant confirms that the ligand adopts a horseshoe-like conformation, forming critical interactions with residues such as GLN126, ASN318, and HIS344 [40,41]. Similarly, the structure of OXR1 bound to suvorexant reveals that the ligand stabilizes the receptor through hydrogen bonds with GLU204 and hydrophobic interactions with PHE220 and PHE340 [42].

### 3.3. Dynamics and Intermolecular Interactions of DORA–OXR1 Complexes

The molecular dynamics simulations provided critical insights into the stability and conformational dynamics of the DORA–OXR1 complexes. Daridorexant and suvorexant exhibited early stabilization, with RMSD values converging within the first 50 ns of the simulation. This rapid stabilization suggests that these ligands quickly adopt a stable conformation within the binding pocket, which may contribute to their high binding affinity and efficacy. In contrast, lemborexant showed pronounced fluctuations during the initial 150 ns of the simulation, indicating a slower conformational convergence.

The RMSF analysis further highlighted the stabilizing effect of the ligand interactions on the OXR1 binding site. The residues in the binding pocket exhibited low fluctuation values (≈0.2 nm), indicating that the ligands effectively stabilized the receptor structure. However, higher fluctuations were observed in extracellular and intracellular loops, as well as in terminal regions, suggesting that these areas retain a degree of flexibility even in the presence of bound ligands. This flexibility may be important for the receptor’s ability to undergo conformational changes necessary for signal transduction. In particular, the transmembrane domains remained stable throughout the 700 ns simulation, highlighting the structural resilience of OXR1 under ligand antagonism.

The interaction energy analysis revealed significant differences in the interaction profiles of the three DORAs with OXR1. The daridorexant exhibited greater variability in the interaction energy across dielectric constants ϵ (10, 20, and 40), suggesting a pronounced sensitivity to electrostatic environmental changes. The observed variability in daridorexant’s interaction energy is likely attributed to its higher dipole moment (3.81 D) and larger cavity volume (3127.75 Å^3^). These properties make its binding interactions more dependent on fluctuations in the local electrostatic potential. Conversely, lemborexant and suvorexant exhibited relatively stable interaction energy profiles under dielectric conditions, indicating lower susceptibility to electrostatic variations. This stability likely arises from stronger hydrophobic interactions or a more consistent charge distribution within their binding sites.

The interaction energy analysis identified key residues contributing to ligand binding within the OXR1 receptor pocket. In the daridorexant–OXR1 complex, GLU204, HIS216, and ASN318 played dominant roles, with GLU204 exhibiting the strongest interaction energy (−25.18 kcal/mol). These residues formed critical hydrogen bonds and electrostatic interactions with key functional groups in daridorexant, such as the amide group and aromatic rings, stabilizing the ligand within the binding site. Specifically, the carbonyl group of GLU204 formed hydrogen bonds with the amine groups of daridorexant, while the imidazole ring of HIS216 engaged in a hydrogen bond with the ligand’s aromatic rings.

In the lemborexant–OXR1 complex, HIS344, VAL130, TYR311, and GLN126 were the primary contributors (−19.28 kcal/mol) to binding stability. HIS344 and GLN126 engaged π–π stacking interactions with the pyrimidine ring of lemborexant, highlighting the contribution of the pyrimidine ring to maintaining hydrophobic interactions with the receptor. Furthermore, the C21 carbon substituent on this ring engages in three hydrophobic interactions: two π–alkyl interactions with HIS344 and TYR311 and one alkyl interaction with HIS344. The VAL130 residue also forms significant hydrophobic interactions with both the pyrimidine and benzene rings. These results highlight the importance of heteroaromatic interaction rings and nonpolar functional groups in stabilizing lemborexant within the binding pocket.

Similarly, in the suvorexant–OXR1 complex, HIS344, GLN126, PRO123, and ASN318 played crucial roles. This group of amino acids establishes hydrophobic interactions and hydrogen bonds (−16.54 kcal/mol) that are important for stabilizing the complex. The interactions that contribute the most energetically are the π–alkyl interactions formed by HIS344—one with the diazepine ring, the other with its methyl substituent. Additionally, PRO123 engages in a π-alkyl interaction with the triazole ring, and GLN126 establishes an alkyl interaction with the diazepine ring. Furthermore, ASN318 engaged in hydrogen bonding with the benzoxazole group of the ligand. These interactions emphasize the critical role of aromatic systems and polar moieties in ligand–receptor binding.

The experimental binding affinity data reveal distinct OXR1 affinity profiles among orexin antagonists. Daridorexant exhibits the highest binding affinity for OXR1 ($K_{i}$ = 0.47 nM) [43,44], followed by suvorexant ($K_{i}$ = 0.55 nM) [45,46], while lemborexant shows a significantly weaker interaction ($K_{i}$ = 6.1 nM) [40,47]. This experimental ranking is consistently replicated by our computational interaction energy results, where daridorexant demonstrated improved thermodynamic stabilization (−83.02 kcal/mol) compared to suvorexant (−52.77 kcal/mol) and lemborexant (−49.87 kcal/mol).

This study’s findings establish a robust molecular basis for understanding the interactions between DORAs and OXR1, offering valuable insights for the rational design of next-generation orexin receptor antagonists, particularly in the hydrophobic subdomains, to achieve pharmacological selectivity.

## 4. Materials and Methods

**Figure 8:**
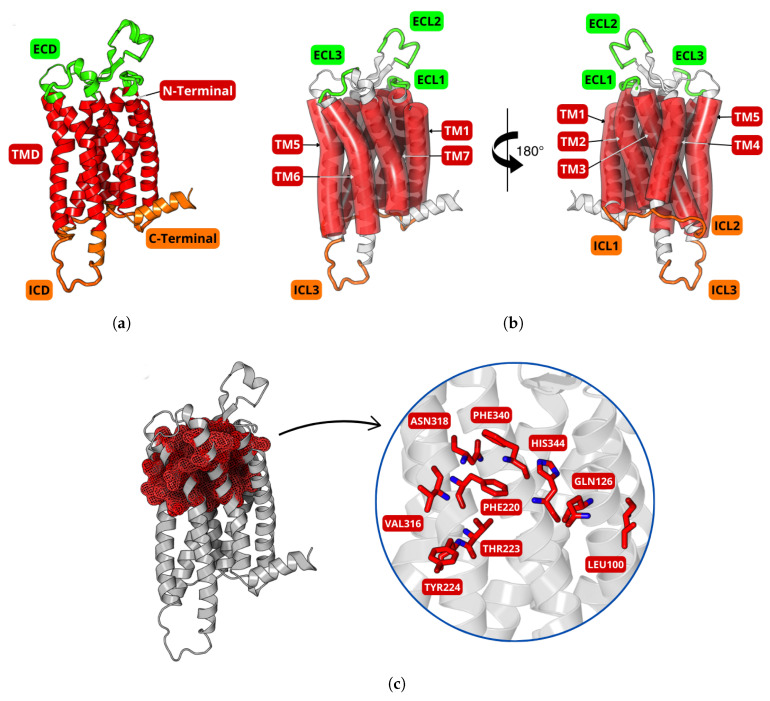
Ribbon depiction of orexin receptor 1 (OXR1) structure. (**a**) The overall arrangement of the transmembrane domains (TMDs) in red, with extracellular (ECD) in green and intracellular (ICD) in orange, focusing on the N-terminal and C-terminal sections. (**b**) The transmembrane α-helices (TM1–TM7), extracellular loops (ECL1–ECL3), and intracellular loops (ICL1–ICL3). (**c**) Mesh surface of the binding site in red, highlighting key residues forming the site.

In this study, the interactions of the dual orexin receptor antagonists daridorexant, lemborexant, and suvorexant with the orexin receptor OXR1 were comprehensively analyzed through an integrated approach involving molecular docking, molecular dynamics simulations, and density functional theory quantum calculations. The three-dimensional structures of the ligands were obtained in SDF format from the PubChem database (https://pubchem.ncbi.nlm.nih.gov/), while the OXR1 receptor structure was retrieved from the RCSB Protein Data Bank (https://www.rcsb.org/, PDB ID: 6TOD) [41]. As illustrated in Figure 8a, this G-protein-coupled receptor (GPCR) comprises an extracellular domain (ECD, green), a transmembrane domain (TMD, red), and an intracellular domain (ICD, orange). The TMD includes seven α-helices (TM1–TM7) arranged in a helical bundle, interconnected by intracellular (ICL1–ICL3) and extracellular loops (ECL1–ECL3), as shown in Figure 8b. Figure 8c highlights the binding site of the OXR1 receptor, shown as a red mesh surface. The main residues include polar/hydrophilic amino acids: L-glutamine (GLN126), L-threonine (THR223), L-tyrosine (TYR224), L-asparagine (ASN318), and L-histidine (HIS344), located, respectively, in the transmembrane α-helices TM3, TM5, TM5, TM6, and TM7; and nonpolar/hydrophobic amino acids: L-leucine (LEU110), L-phenylalanine (PHE340 and PHE340), and L-valine (VAL317), located. respectively, in the transmembrane α-helices TM2, TM5, TM7, and TM6, suggesting important roles in the interaction of ligands.

### 4.1. Ligand Molecular Optimization and DFT Calculation

Ligand geometries were initially optimized using a Dreiding-like force field, followed by a two-step minimization with the CHARMm force field (version c44b2) and the SmartMinimizer algorithm, under a distance-dependent dielectric environment. All procedures were performed in BIOVIA Discovery Studio (https://www.3ds.com/products/biovia/discovery-studio, accessed on 26 May 2025). To explore conformational flexibility, the GenerateConformations function (BEST method) was applied, which combines torsion-space minimization, Cartesian-space optimization, and quasi-Newton refinement. The Poling algorithm and Boltzmann jump method were used to generate energetically favorable and diverse conformers.

Quantum chemical calculations based on DFT with the GGA-PBE functional and 6-311++G basis set [36] were employed for detailed geometry optimizations and electronic structure analyses [48,49]. The electronic characterization included parameters such as total energy, cohesive energy, HOMO–LUMO energies and gap, dipole moment, dielectric and solvation energies, solvent-accessible surface area, and cavity volume. Atomic charges were evaluated using ESP, Mulliken, and Hirshfeld methods [50]. These calculations were also performed using Discovery Studio.

In addition, quantum descriptors such as ionization potential (I), electron affinity (A), chemical hardness (η), softness (σ), chemical potential (μ), electronegativity (χ), and electrophilicity index (ω) were analyzed using validated protocols [51,52]. These parameters provide key insights into ligand stability and reactivity within receptor binding sites [53].

### 4.2. Protein Preparation and Molecular Docking

All heteroatoms and water molecules were removed from the protein structure (PDB ID: 6TOD) and saved as pdb format. Subsequently, the structures that contained gaps in the amino acid sequence were modeled by homology using Modeller software [54] version 10.4 (https://salilab.org/modeller/, accessed on 26 May 2025) and confirmed using Alphafold2 software (ColabFold v1.5.5) (https://colab.research.google.com/github/sokrypton/ColabFold/blob/main/AlphaFold2.ipynb, accessed on 26 May 2025) [55]. To predict the preferred conformations of the ligand molecules and the receptor, molecular docking procedures were performed using the High Ambiguity Driven Biomolecular DOCKing (HADDOCK 2.4—https://wenmr.science.uu.nl/haddock2.4/, accessed on 26 May 2025) [56,57]. This procedure enabled prediction of how the ligands interact with the proteins and, using the same server, determination of scoring metrics (HADDOCK score, HS) to select the complex models with the most energetically favorable interactions between the two parts [58]. The HADDOCK score consists of a linear combination involving terms referring to the relative energies of van der Waals interactions ($E_{\mathrm{VWD}}$), Coulombian electrostatic interactions ($E_{\mathrm{ELEC}}$), desolvation ($E_{\mathrm{DESOLV}}$), and restraints ($E_{\mathrm{AIR}}$), according to Equation (1). The complex with the lowest energy was then used as the initial structure for molecular dynamics simulations.

(1) $$\mathrm{HS}=1.0\cdot E_{\mathrm{VWD}}+0.2\cdot E_{\mathrm{ELEC}}+1.0\cdot E_{\mathrm{DESOLV}}+0.1\cdot E_{\mathrm{AIR}}$$

### 4.3. Molecular Dynamics Simulation

**Figure 9:**
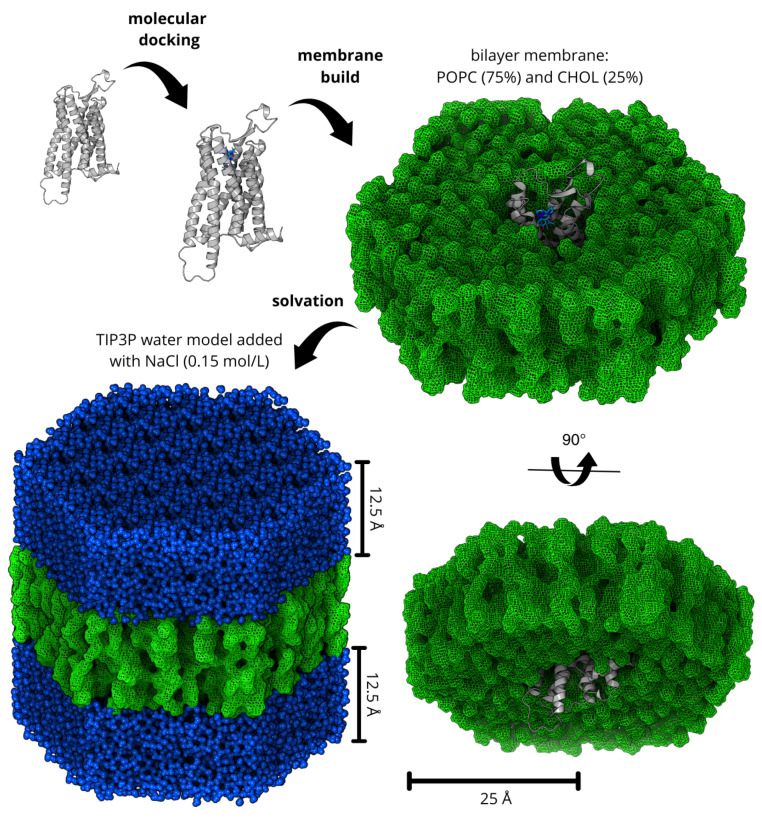
Graphical illustration of the step-by-step process for obtaining a solvated transmembrane receptor system with a docked drug. The lipid bilayer, composed of POPC (75%) and cholesterol (25%), is represented as a green mesh surface, while the receptor is depicted in a ribbon representation. The final system is fully solvated, with the hydration layer shown in blue spheres, consisting of TIP3P water molecules and Na^+^ and Cl^−^ ions at a concentration of 0.15 mol/L.

Molecular dynamics simulations were performed using GROMACS software (https://www.gromacs.org/) [59], version 2023.1, with the CHARMM36 force field [60,61]. The server CHARMM-GUI (https://www.charmm-gui.org/) [62,63,64] was used to generate hexagonal bilayer membranes solvated in the Transferable Intermolecular Potential 3 Point (TIP3P) water model [65]. The membrane comprised 168 lipids for monolayer, 126 POPC, 1-palmitoyl-2-oleoylphosphatidyl-choline (75%), and 42 CHOL, cholesterol (25%). The membrane dimensions were selected to maintain at least 50 Å separation between periodic images in the membrane plane and 25 Å perpendicular to it [37]. ${\mathrm{Na}}^{+}$ and ${\mathrm{Cl}}^{-}$ ions were added to neutralize the system, achieving a concentration of 0.15 mol/L. These procedures are depicted in Figure 9.

The system was maintained at a pH of 7.40. The equations of motion were integrated using the Leap-Frog algorithm [66] with a 2.0 fs integration time step. Long-range interactions were handled using the Particle Mesh Ewald sum (PME) method [67] with a cutoff of 1.2 nm, including van der Waals interactions. Covalent bonds involving hydrogen atoms were constrained using the Linear Constraint Solver for Molecular Simulations (LINCS) algorithm [68]. The geometry of the system was minimized using the steepest descent algorithm [69] over 10,000 steps, with a tolerance of 10 kJ mol^−1^ nm^−1^. The system was equilibrated in two 1 ns phases: first under the NVT ensemble (constant volume and temperature), followed by the NPT ensemble (constant pressure and temperature). The production dynamics were carried out over 700 ns of simulation time (for each complex), resulting in 2.1 μs of total trajectories (all complexes). V-rescale thermostat [70] was used to maintain the temperature at 310.14 K, while the Parrinello–Rahman barostat [71] controlled the pressure in the NPT ensembles.

Molecular dynamics simulations were employed to assess the conformational stability of the protein–ligand complexes. The evaluation included calculating the RMSD of the protein backbone α-carbon atoms and the RMSF of individual residues to determine the overall stability and flexibility of the proteins. Furthermore, B-factors (Debye–Waller factors) were examined to measure residue mobility within the protein structure. The interaction analysis encompassed intermolecular hydrogen bonds (conventional, nonconventional, carbon–hydrogen, π-donor, and H-bonds), electrostatic interactions (attractive charges, salt bridges, π–cation, and π–anion), hydrophobic contacts (π–π stacked, π–π T stacked, amide–π stacked, alkyl, π–sigma, and π–alkyl), halogen bonds (F, Cl, Br, and I), as well as miscellaneous interactions (steric clashes, charge repulsion, acceptor–acceptor collisions, and metal repulsion), including unfavorable interactions [72]. Furthermore, three-dimensional conformational representations of the protein–ligand complexes were generated using BIOVIA Discovery Studio software, version 2022, with detailed depictions of the protein–ligand interactions.

All images showing ligand–protein complex interactions were produced using BIOVIA Discovery Studio software and the 3D Protein Imaging server (https://3dproteinimaging.com/). Graphical representations were generated using the tools for Graphing, Advanced Computation, and Data Exploration, Grace (https://plasma-gate.weizmann.ac.il/Grace/, accessed on 26 May 2025), and Scientific Data Analysis and Visualization, SciDAVis (https://scidavis.sourceforge.net/index.html, accessed on 26 May 2025).

### 4.4. Molecular Fractionation with Conjugate Cap

Molecular fractionation with conjugate cap technique was applied to accurately compute the interaction potential energies between the ligands and specific amino acid residues of the OXR1 receptor based on the stable conformations obtained from extensive 700 ns molecular dynamics simulations. The MFCC approach, originally developed by DW Zhang and Zhang [73], is particularly well suited to investigate quantum mechanical interactions in complex biomolecular systems, offering a reliable balance between computational cost and accuracy [74,75,76,77,78]. This methodology sections the protein–ligand complex into chemically relevant fragments, where each amino acid fragment is separated by cleaving peptide bonds and capped with conjugated groups to ensure that local chemical environments and valence requirements are maintained. In this case, the ligands are denoted as *L*, and the interacting residue is identified as $R^{i}$, where *i* indicates the index of the specific residue. The energy of interaction between the OXR1 residues and the ligands, $EI(L-R^{i})$, is calculated following the standard protocol described in Equation (2).

(2) $$EI\left(L-R^{i}\right)=E\left(L-C^{i-1}R^{i}C^{i+1}\right)-E\left(C^{i-1}R^{i}C^{i+1}\right)-E\left(L-C^{i-1}C^{i+1}\right)+E\left(C^{i-1}C^{i+1}\right)$$

In this equation, $C^{i-1}$ and $C^{i+1}$ represent the neighboring residues (caps) attached to the reference residue $R^{i}$. The term $E\left(L-C^{i-1}R^{i}C^{i+1}\right)$ denotes the total energy of the system comprising the ligand and the capped residue. By subtracting the energy of the capped residue, $E\left(C^{i-1}R^{i}C^{i+1}\right)$, and the energy of the system formed by the ligand and the caps, $E\left(L-C^{i-1}C^{i+1}\right)$, we obtain the ligand–residue interaction energy. The energy of interaction between the caps is subtracted twice, and, to correct for this, we add the term $E\left(C^{i-1}C^{i+1}\right)$. To preserve molecular integrity, hydrogen atoms were added to the caps to eliminate dangling bonds, thus stabilizing each fragment’s structure for individual calculations.

### 4.5. Quantum Calculations

To ensure an accurate characterization of intermolecular interactions and binding affinities in systems, this study employed a hybrid computational approach that integrates classical and quantum methods [76,77,78,79]. Following simulations, the ligand–OXR1 complexes were fragmented using the MFCC approach. The resulting structures were then subjected to quantum mechanical energy calculations within the DFT formalism [80] through the Gaussian 16 package [81]. To enhance the representation of non-covalent interactions, the B97D functional, a generalized gradient approximation (GGA) [82] class functional with dispersion corrections, was applied. The Kohn–Sham orbitals were detailed using the 6-311+G(d,p) basis set for precise electronic interaction characterization. To account for the influence of neighboring residues and solvent molecules lost during fragmentation, the calculations incorporated a polarizable continuum model (CPCM) [83] using dielectric constants of ϵ= 10, 20, and 40. These values simulate a range of electrostatic environments, enabling a more accurate evaluation of binding interactions within the biological system.

To ensure that all relevant interactions between the ligands and binding site residues were accounted for, a convergence analysis of the total interaction energy was conducted as a function of the ligand binding pocket radius (r), ranging from 2.0 to 8.0 Å. This approach systematically defines the binding pocket by progressively incorporating amino acid residues based on their distance from the ligand. Imaginary spheres with increasing radii (r = n/2; n = 1, 2, 3, …) were used to delineate these distances, summing the interaction energies of residues within each sphere. Convergence was reached when the relative energy difference between successive iterations fell below 10% [75].

## 5. Conclusions

This study employed a comprehensive multiscale computational approach to elucidate the binding mechanisms and interaction profiles of three OXR1 antagonists—daridorexant, lemborexant, and suvorexant. By integrating 700 ns molecular dynamics simulations with molecular fractionation using conjugate caps (MFCC) and density functional theory (DFT) calculations, we obtained detailed insights into their thermodynamic stabilization and structural dynamics within the receptor binding site.

The comparative analysis revealed that daridorexant exhibited the most favorable interaction energy profile (−83.02 kcal/mol), followed by suvorexant (−52.77 kcal/mol) and lemborexant (−49.87 kcal/mol). This ranking was consistent with the experimental binding affinity $K_{i}$ values (daridorexant = 0.47 nM > suvorexant = 0.55 nM > lemborexant = 6.1 nM), supporting the predictive accuracy of our computational pipeline. Daridorexant’s superior binding was associated with favorable interactions involving key residues—particularly GLU204, HIS216, and ASN318—mediated by a synergistic combination of hydrogen bonding and hydrophobic contacts. In contrast, lemborexant and suvorexant were primarily stabilized by hydrophobic interactions.

In conclusion, this work advances the mechanistic understanding of OXR1 inhibition and highlights the effectiveness of integrated computational strategies in accurately predicting drug–receptor interactions. These results lay the groundwork for the rational optimization of lead compounds targeting sleep disorders and related neuropsychiatric conditions.

## Figures and Tables

**Table 1:** The quantum descriptors: GAP energy (HOMO–LUMO), ionization potential (*I*), electron affinity (*A*), chemical hardness (η), softness (σ), chemical potential (μ), electronegativity index (χ), and electrophilicity index (ω). All values in eV, unless specified. Softness (σ) is reported in eV^−1^.

DORA	GAP	*I*	*A*	η	σ	μ	χ	ω
Daridorexant	4.86721	6.19316	1.32595	2.43360	0.41091	3.75956	−3.75956	17.19860
Lemborexant	5.42455	6.53966	1.11510	2.71228	0.36869	3.82738	−3.82738	19.84586
Suvorexant	4.47390	5.87751	1.40361	2.23695	0.44704	3.64056	−3.64056	14.82392

**Table 2:** Main data obtained from the scoring process performed after molecular docking for each DORA–OXR1 complex: number of structures obtained (cluster size), HADDOCK score (HS, in kcal/mol), root-mean-square deviation (RMSD, in Å), relative energies of van der Waals interactions (E_VDW_, in kcal/mol) and Coulombic electrostatic interactions (E_ELEC_, in kcal/mol), and Z-score of the structure with the lowest HS.

DORA	Cluster Size	HS	RMSD	E_VDW_	E_ELEC_	Z-Score
Daridorexant	199	−44.8 ± 0.9	0.2 ± 0.1	−25.2 ± 4.0	−59.6 ± 16.7	0.0
Lemborexant	200	−39.9 ± 1.0	0.3 ± 0.2	−23.2 ± 2.3	−33.1 ± 15.6	0.0
Suvorexant	200	−43.7 ± 1.7	0.3 ± 0.2	−25.2 ± 0.7	−53.0 ± 10.3	0.0

## Data Availability

Data will be made available on request.

## Abbreviations

The following abbreviations are used in this manuscript:

OXR1	Orexin A receptor
OXR2	Orexin B receptor
DORA	Dual orexin receptor antagonist
MD	Molecular dynamics
MFCC	Molecular fractionation with conjugate caps
DFT	Density functional theory
SASA	Solvent-accessible surface area
RMSD	Root-mean-square deviation
MO	Molecular orbitals
HOMO	Highest occupied molecular orbital
LUMO	Lowest unoccupied molecular orbital
RMSF	Root-mean-square fluctuation
GPCR	G-protein-coupled receptor
